# Microbial Cellulases and Their Industrial Applications

**DOI:** 10.4061/2011/280696

**Published:** 2011-09-07

**Authors:** Ramesh Chander Kuhad, Rishi Gupta, Ajay Singh

**Affiliations:** ^1^Lignocellulose Biotechnology Laboratory, Department of Microbiology, University of Delhi South Campus, New Delhi 110021, India; ^2^Research & Development Division, Lystek International Inc., Waterloo, ON, N2J 3H8, Canada

## Abstract

Microbial cellulases have shown their potential application in various industries including pulp and paper, textile, laundry, biofuel production, food and feed industry, brewing, and agriculture. Due to the complexity of enzyme system and immense industrial potential, cellulases have been a potential candidate for research by both the academic and industrial research groups. Nowadays, significant attentions have been devoted to the current knowledge of cellulase production and the challenges in cellulase research especially in the direction of improving the process economics of various industries. Scientific and technological developments and the future prospects for application of cellulases in different industries are discussed in this paper.

## 1. Introduction

Biotechnological conversion of cellulosic biomass is potentially sustainable approach to develop novel bioprocesses and products. Microbial cellulases have become the focal biocatalysts due to their complex nature and wide spread industrial applications. Cellulases are composed of independently folding, structurally and functionally discrete units called domains or modules, making cellulases module [1]. Cellulases are inducible enzymes synthesized by a large diversity of microorganisms including both fungi and bacteria during their growth on cellulosic materials (Table 1) [2, 3]. These microorganisms can be aerobic, anaerobic, mesophilic or thermophilic. Among them, the genera of *Clostridium, Cellulomonas, Thermomonospora, Trichoderma, *and* Aspergillus *are the most extensively studied cellulase producer [4–7]. 

Structurally fungal cellulases are simpler as compared to bacterial cellulase systems, cellulosomes [8–10]. Fungal cellulases typically have two separate domains: a catalytic domain (CD) and a cellulose binding module (CBM), which is joined by a short polylinker region to the catalytic domain at the N-terminal. The CBM is comprised of approximately 35 amino acids, and the linker region is rich in serine and threonine. The main difference between cellulosomes and free cellulase enzyme is in the component of cellulosomes-cohesin containing scaffolding and dockerin containing enzyme. The free cellulase contains cellulose binding domains (CBMs), which are replaced by a dockerin in cellulosomal complex, and a single scaffolding-born CBM directs the entire cellulosomes complex to cellulosic biomass [11, 12].

Mechanistically, cellulase is a family of at least 3 groups of enzymes [10, 13–15], endo-(1,4)-*β*-D-glucanase (EC 3.2.1.4) exo-(1,4)-*β*-D-glucanase (EC 3.2.1.91), and *β*-glucosidases (EC 3.2.1.21). The exoglucanase (CBH) acts on the ends of the cellulose chain and releases *β*-cellobiose as the end product; endoglucanase (EG) randomly attacks the internal *O*-glycosidic bonds, resulting in glucan chains of different lengths; and the *β*-glycosidases act specifically on the *β*-cellobiose disaccharides and produce glucose [8, 16]. Although the mechanism of cellulose degradation by aerobic bacteria is similar to that of aerobic fungi, it is clear that anaerobic bacteria operate on a different system [10, 11]. Cellulosomes located on the cell surface mediate adherence of anaerobic cellulolytic bacteria to the substrate, which thereafter undergo a supramolecular reorganization, so that the cellulosomal subunits redistribute to interact with the different target substrates [12].

Cellulases have been commercially available for more than 30 years, and these enzymes have represented a target for both academic as well as industrial research [16, 17]. Basic and applied studies on cellulolytic enzymes have demonstrated their biotechnological potential in various industries including food, animal feed, brewing and wine making, agriculture, biomass refining, pulp and paper, textile, and laundry. In the present paper, the potent industrial applications of cellulases have been critically reviewed.

## 2. Application of Cellulases in Various Industries

Microbial cellulases find applications in various industries as shown in Table 2.

### 2.1. Pulp and Paper Industry

Interest in the application of cellulases in the pulp and paper industry has increased considerably during the last decade [18]. The mechanical pulping processes such as refining and grinding of the woody raw material lead to pulps with high content of fines, bulk, and stiffness. While in contrast, biomechanical pulping using cellulases resulted in substantial energy savings (20–40%) during refining and improvements in hand-sheet strength properties [17, 19–21]. 

Mixtures of cellulases (endoglucanases I and II) and hemicellulases have also been used for biomodification of fiber properties with the aim of improving drainage and beatability in the paper mills before or after beating of pulp [22]. Mansfield et al. [23] studied the action of a commercial cellulase preparation on different fractions of Douglas fir kraft pulp and observed that the cellulase treatment decreased the defibrillation reducing the fibre coarseness. While endoglucanases have the ability to decrease the pulp viscosity with a lower degree of hydrolysis [24], cellulases have also been reported to enhance the bleachability of softwood kraft pulp producing a final brightness score comparable to that of xylanase treatment [17, 25].

Cellulases alone, or used in combination with xylanases, are beneficial for deinking of different types of paper wastes. Most applications proposed so far use cellulases and hemicellulases for the release of ink from the fiber surface by partial hydrolysis of carbohydrate molecules [26]. It has been postulated that improvements in dewatering and deinking of various pulps result in the peeling of the individual fibrils and bundles, which have high affinity for the surrounding water and ink particles [27]. The main advantages of enzymatic deinking are reduced or eliminated alkali usage, improved fiber brightness, enhanced strength properties, higher pulp freeness and cleanliness, and reduced fine particles in the pulp [26, 28]. Moreover, deinking using enzymes at acidic pH also prevents the alkaline yellowing, simplifies the deinking process, changes the ink particle size distribution, and reduces the environmental pollution. Although enzymatic deinking can lower the need for deinking chemicals and reduce the adverse environmental impacts of the paper industry [29], the excessive use of enzymes must be avoided [29], because significant hydrolysis of the fines could reduce the bondability of the fibers [30]. 

Interestingly, the use of cellulases in improving the drainage has also been pursued by several mills with the objective to increase the production rate. Enzyme treatments remove some of the fines or peel off fibrils on the fiber surface and dissolved and colloidal substances, which often cause severe drainage problems in paper mills. In this aspect, cellulases have shown considerable improvement in the overall performance of paper mills [21, 31]. Enzymatic treatment also destabilizes the lipophilic extractives in the filtrates and facilitates their attachment to thermomechanical pulping fibers. These enzymes are also used in preparation of easily biodegradable cardboard [32], manufacturing of soft paper including paper towels and sanitary paper [33, 34], and removal of adhered paper [35].

### 2.2. Textile Industry

Cellulases are the most successful enzymes used in textile wet processing, especially finishing of cellulose-based textiles, with the goal of improved hand and appearance [36, 37]. Traditional stonewashing of jeans involves amylase-mediated removal of starch coating (desizing) and treatment (abrasion) of jeans with pumice stone (1-2 kg/pair of jeans) in large washing machines. Cellulases have been successfully used for the biostoning of jeans and biopolishing of cotton and other cellulosic fabrics. During the biostoning process, cellulases act on the cotton fabric and break off the small fiber ends on the yarn surface, thereby loosening the dye, which is easily removed by mechanical abrasion in the wash cycle. The advantages in the replacement of pumice stones by a cellulose-based treatment include less damage of fibers, increased productivity of the machines, and less work-intensive and environment benign [5, 17, 38, 39]. 

While the biopolishing is usually carried out during the wet processing stages, which include desizing, scouring, bleaching, dyeing, and finishing. The acidic cellulases improve softness and water absorbance property of fibres, strongly reduce the tendency for pill formation, and provide a cleaner surface structure with less fuzz [40]. Cellulase preparations rich in endoglucanases are best suited for biopolishing enhancing fabric look, feel, and color without needing any chemical coating of fibers [39]. The action of cellulases removes short fibers, surface fuzziness, creates a smooth and glossy appearance, and improves color brightness, hydrophilicity and moisture absorbance, and environmentally friendly process [21]. 

Similarly, endoglucanase activity-rich cellulase is also proved better for biofinishing. Most cotton or cotton-blended garments, during repeated washing, tend to become fluffy and dull, which is mainly due to the presence of partially detached microfibrils on the surface of garments. The use of cellulases can remove these microfibrils and restore a smooth surface and original color to the garments [36, 41]. The use of cellulase also helps in softening the garments and in removal of dirt particles trapped within the microfibril network. 

Interestingly, there are several reports where the performance of the whole cellulase preparations was quite different from the enzyme rich in endoglucanase activity, and that the latter offered better performance in applications where losses in fabric strength and weight were minimum. Depilling/cleaning and/or ageing effects are the result of the synergistic action of cellulases and mechanical action, simultaneously or sequentially [42]. Attempts have also been made via cellulases treatment to improve the dimensional stability of cellulosic fabrics and to upgrade the surface and dyeing properties of bleached cotton, mercerized cotton, and cotton/polyester blend fabric (50/50), using the pad-wet batch technique, followed by subsequent washing under mechanical action [41, 43].

### 2.3. Bioethanol Industry

Enzymatic saccharification of lignocellulosic materials such as sugarcane bagasse, corncob, rice straw, *Prosopis juliflora*,* Lantana camara*, switch grass, saw dust, and forest residues by cellulases for biofuel production is perhaps the most popular application currently being investigated [5, 6, 44]. Bioconversion of lignocellulosic materials into useful and higher value products normally requires multistep processes [6, 45, 46]. These processes include; pretreatment (mechanical, chemical, or biological), hydrolysis of the polymers to produce readily metabolizable molecules (e.g., hexose and pentose sugars), bioconversion of these smaller molecules to support microbial growth and/or produce chemical products, and the separation and purification of the desired products. The utility cost of enzymatic hydrolysis may be low compared with acid or alkaline hydrolysis because enzyme hydrolysis is usually conducted at mild conditions (pH 4–6 and temperature 45–50°C) and does not have corrosion issues [26, 44].

Technologies are currently available for all steps in the bioconversion of lignocellulosics to ethanol and other chemical products [4, 13, 47, 48]. However, some of these technologies must be improved to produce renewable biofuel and other byproducts at prices, which can compete with more conventional production systems. Not only the recalcitrance of the substrate, but also several other factors that also limit cellulase efficiency during the hydrolysis process including end product inhibition, thermal deactivation of the native protein, nonspecific binding to lignin [49], and irreversible adsorption of the enzymes to the heterogeneous substrate [50].

To reduce the enzyme cost in the production of fuel ethanol from lignocellulosic biomass, two aspects are widely addressed: optimization of the cellulase production and development of a more efficient cellulase-based catalysis system. Protein engineering and directed evolution are powerful tools that can facilitate the development of more efficient thermophilic cellulases [42]. Strategies for recycling and reusage of the enzymes may also be used to reduce enzymatic hydrolysis costs [4, 48, 51, 52]. The recovery of enzymes is largely influenced by adsorption of the enzymes onto the substrate, especially to lignin and enzyme inactivation. There are several reports where the nonspecific and irreversible adsorption of cellulase to lignin has been observed [53, 54]. Besides, there are also reports where the compounds that mimic cellulose or the compounds have high affinity towards lignin have been used to prevent the adsorption of cellulases to lignin [55, 56]. Moreover, recently Scott and coworkers [57] have filed a US patent (20100221778) on novel lignin-resistant cellulase enzyme, in which linker peptides have been modified to prevent their adsorption onto lignin and enhance the enzyme activity. Among different strategies to recover and reuse the cellulases are concentration of the cellulose fraction by ultrafiltration to remove sugars and other small compounds that may inhibit the action of the enzymes [58] and recycling of immobilized enzymes, which enables separation of the enzymes from the process flow [48, 59]. However, the recycling techniques are mostly tested at laboratory scale. Therefore, the ability to scale up the techniques, the robustness, and feasibility still needs to be demonstrated.

### 2.4. Wine and Brewery Industry

Microbial glucanases and related polysaccharides play important roles in fermentation processes to produce alcoholic beverages including beers and wines [5, 17, 39, 60]. These enzymes can improve both quality and yields of the fermented products [60]. Glucanases are added either during mashing or primary fermentation to hydrolyze glucan, reduce the viscosity of wort, and improve the filterability [60, 61].

In wine production, enzymes such as pectinases, glucanases, and hemicellulases play an important role by improving color extraction, skin maceration, must clarification, filtration, and finally the wine quality and stability [17, 39]. *β*-Glucosidases can improve the aroma of wines by modifying glycosylated precursors. Macerating enzymes also improve pressability, settling, and juice yields of grapes used for wine fermentation. A number of commercial enzyme preparations are now available to the wine industry. The main benefits of using these enzymes during wine making include better maceration, improved color extraction, easy clarification, easy filtration, improved wine quality, and improved stability [39]. 

Beer brewing is based on the action of enzymes activated during malting and fermentation. Malting of barley depends on seed germination, which initiates the biosynthesis and activation of *α*- and *β*-amylases, carboxypeptidase, and *β*-glucanase that hydrolyze the seed reserves [60]. In an earlier study, Oksanen et al. [62] observed that endoglucanase II and exoglucanase II of the *Trichoderma *cellulase system were responsible for a maximum reduction in the degree of polymerization and wort viscosity. 

Significant and reproducible improvements in grape pressability, settling rate, and total juice yield were achieved using a combination of macerating enzymes. Such improvements were noticeable only with a correct balance of pectinases, cellulases, and hemicellulases. Using three varieties (Soave, Chardonnay, and Sauvignon) of white grapes, Galante et al. [39] assessed the performance of Cytolase 219 (mixture of cellulase, pectinase, and xylanase) in wine making and reported a 10–35% increase in the extraction of the first wine must, a 70–80% increase in the must filtration rate, 50–120 minutes decrease in pressing time, 30–70% decrease in must viscosity, 20–40% saving of energy during cooling of fermenter, and a significant improvement in wine stability. A range of improved enzymes like cellulase and pectinase that would be exogenously added to the process are expected to enhance the productivity of existing brewing processes in future [60].

### 2.5. Food Processing Industry

Cellulases have a wide range of potential applications in food biotechnology as well. The production of fruit and vegetable juices requires improved methods for extraction, clarification, and stabilization. Cellulases also have an important application as a part of macerating enzymes complex (cellulases, xylanases, and pectinases) used for extraction and clarification of fruit and vegetable juices to increase the yield of juices [63, 64]. The use of macerating enzymes increases both yield and process performance without additional capital investment. The macerating enzymes are used to improve cloud stability and texture and decrease viscosity of the nectars and purees from tropical fruits such as mango, peach, papaya, plum, apricot, and pear [5, 17, 21, 64]. Texture, flavor, and aroma properties of fruits and vegetables can be improved by reducing excessive bitterness of citrus fruits by infusion of enzymes such as pectinases and *β*-glucosidases [65–67]. Enzyme mixtures containing pectinases, cellulases, and hemicellulases are also used for improved extraction of olive oil. Use of macerating enzymes not only improves the cloud stability and texture of nectars and purees, but also decreases their viscosity rapidly [68]. Thus, the macerating enzymes, composed of mainly cellulase and pectinase, play a key role in food biotechnology, and their demand will likely increase for extraction of juice from a wide range of fruits and vegetables [59]. Furthermore, infusion of pectinases and *β*-glucosidases has also shown to alter the texture, flavor, and other sensory properties such as aroma and volatile characteristics of fruits and vegetables [17, 21, 37, 69, 70].

### 2.6. Animal Feed Industry

Applications of cellulases and hemicellulases in the feed industry have received considerable attention because of their potential to improve feed value and performance of animals [71]. Pretreatment of agricultural silage and grain feed by cellulases or xylanases can improve its nutritional value [72]. The enzymes can also eliminate antinutritional factors present in the feed grains, degrade certain feed constituents to improve the nutritional value, and provide supplementary digestive enzymes such as proteases, amylases, and glucanases. For instance, the dietary fiber consists of nonstarch polysaccharides such as arabinoxylans, cellulose, and many other plant components including resistant dextrins, inulin, lignin, waxes, chitins, pectins, *β*-glucan, and oligosaccharides, which can act as anti-nutritional factor for several animals such as swine [73] (http://www.nal.usda.gov/fnic/DRI//DRI_Energy/339-421.pdf). In this case, the cellulases effectively hydrolyse the anti-nutritional factor, cellulose, in the feed materials into easily absorbent ingredient thus improve animal health and performance [74] http://www.kdnbiotech.com/en/product_list.aspx?id=10101.

*β*-Glucanases and xylanases have been used in the feed of monogastric animals to hydrolyze nonstarch polysaccharides such as *β*-glucans and arabinoxylans. Cellulases, used as feed additives alone or with proteases, can significantly improve the quality of pork meat. Glucanases and xylanases reduce viscosity of high fibre rye- and barley-based feeds in poultry and pig. These enzymes can also cause weight gain in chickens and piglets by improving digestion and absorption of feed materials [17, 21, 37, 75, 76]. 

Most low-quality feedstuffs contain higher concentrations of cellulose, small amounts of protein and fat, and relatively high ash contents when compared with high-quality feedstuffs. Cellulases can be used to improve silage production for cattle feeding, which involves enhancement of the digestibility of grasses containing large amounts of potentially total digestible nutrients and energy values together with only small amounts of water-soluble carbohydrates. The forage diet of ruminants, which contains cellulose, hemicellulose, pectin, and lignin, is more complex than the cereal-based diet of poultry and pigs. Enzyme preparations containing high levels of cellulase, hemicellulase, and pectinase have been used to improve the nutritive quality of forages [77, 78]. Nevertheless, the results with the addition of enzyme preparations containing cellulase, hemicellulase and pectinase to ruminant diet are somewhat inconsistent.

Animal feedstock production processes generally include heat treatments that inactivate potential viral and microbial contaminants. Application of thermophilic cellulase in feedstock production has the potential to reduce pathogens as well as to enhance digestibility and nutrition of the feed, thereby facilitating a combination of heat treatment and feed transformation in a single step [21]. The cellulases and hemicellulases are responsible for partial hydrolysis of lignocellulosic materials, dehulling of cereal grains, hydrolysis of *β*-glucans, and better emulsification and flexibility of feed materials, which results in the improvement in the nutritional quality of animal feed [39–80]. Moreover, these enzymes can cause partial hydrolysis of plant cell wall during silage and fodder preservation.

The use of enzymes in animal nutrition became more important after the prohibition of using some nutritive ionophore antibiotics, which were previously used in the EU countries [73]. There is a large variation in the digestibility of starch origins. The low digestibility of some starches promotes the appearance of some digestive tract diseases because the nondigested and nonabsorbed starch reaching the large intestine can act as a substrate for bacterial fermentation supporting the proliferation of some potentially hazardous pathogenic bacteria [81, 82]. Cellulases have a positive effect on the caecal fermentation processes by increasing the production of propionic acid, which act as a bacteriostatic material and thus can decrease the colonization of pathogenic bacteria [81, 83].

### 2.7. Agricultural Industries

Various enzyme preparations consisting of different combinations of cellulases, hemicellulases, and pectinases have potential applications in agriculture for enhancing growth of crops and controlling plant diseases [21, 84]. Plant or fungal protoplasts produced using microbial hydrolases can be used to produce hybrid strains with desirable properties. Cellulases and related enzymes from certain fungi are capable of degrading the cell wall of plant pathogens in controlling the plant disease [21]. Fungal *β*-glucanases are capable of controlling diseases by degrading cell walls of plant pathogens. Many cellulolytic fungi including *Trichoderma *sp., *Geocladium *sp., *Chaetomium *sp., and *Penicillium *sp. are known to play a key role in agriculture by facilitating enhanced seed germination, rapid plant growth and flowering, improved root system and increased crop yields [85–87]. Although these fungi have both direct (probably through growth-promoting diffusible factor) and indirect (by controlling the plant disease and pathogens) effects on plants [85, 86], it is not yet clear how these fungi facilitate the improved plant performance. It has been reported that *β*-1,3-glucanase and N-acetyl-glucosaminidase from *T. harzianum *strain P1 synergistically inhibited the spore germination and germ tube elongation of *B. cinerea *[88]. Moreover, the exoglucanase promoters of *Trichoderma *are used for the expression of the different proteins, enzymes, and antibodies in large amount. The exoglucanase promoters of *Trichoderma* have been used for the expression of chymosin [89] and other proteins: glucoamylase, lignin peroxidase, and laccase [90–92].

Cellulases have also been used for the improvement of the soil quality. Traditionally straw incorporation is considered an important strategy to improve soil quality and reduce dependence on mineral fertilizers [93, 94]. Many studies have attempted to hasten straw decomposition via microbial routes. Cellulolytic fungi applications such as *Aspergillus, Chaetomium*, and *Trichoderma*, [95, 96], and actinomycetes [97] have shown promising results. Fontaine et al. [98] showed that exogenous cellulase supplementation accelerated decomposition of cellulose in soil. Therefore, using exogenous cellulase may be a potential means to accelerate straw decomposition and increase soil fertility [99].

### 2.8. Olive Oil Extraction

In recent years, extraction of olive oil has attracted the interest of international market because of its numerous health claims. Extraction of olive oil involves (1) crushing and grinding of olives in a stone or hammer mill; (2) passing the minced olive paste through a series of malaxeurs and horizontal decanters; (3) high-speed centrifugation to recover the oil [39]. To produce high-quality olive oil, freshly picked, clean, and slightly immature fruits have been used under cold pressing conditions [39, 100]. However, high yields have been obtained with fully ripened fruit, when processed at higher than ambient temperatures, but this resulted in oil with high acidity, rancidity, and poor aroma [39]. Hence, an improved method for the extraction of high-quality olive oil was needed to meet the growing consumer demand. The commercial enzyme preparation, Olivex (a pectinase preparation with cellulase and hemicellulase from *Aspergillus aculeatus*), was the first enzyme mixture used to improve the extraction of olive oil [101]. Furthermore, the use of macerating enzymes increased the antioxidants in extravirgin olive oil and reduced the induction of rancidity [39]. The main advantages of using macerating enzymes during olive oil extraction are (1) increased extraction (up to 2 kg oil per 100 kg olives) under cold processing conditions; (2) better centrifugal fractionation of the oily must; (3) oil with high levels of antioxidants and vitamin E; (4) slow induction of rancidity; (5) overall improvement in plant efficiency; (6) low oil content in the waste water [39]. Likewise, the macerating enzymes could play a prominent role in the extraction of oils from other agricultural oilseed crops.

These enzymes can also be used during olive paste malaxation. The presence of collateral activities of a cellulase and hemicellulase nature of the enzymatic formulation guarantees a rapid and intense disintegration of the cell walls and membranes of the olive fruits, thereby favoring the passage of noble substances (particularly the polyphenols and aromatic precursors) into the final product. It is also used to decrease olive paste viscosity in olive oil production and to intensify the process of extracting the polyphenolic substances contained in the olive fruit [102]. It is necessary to underline that the selected enzymes are naturally present inside the olive fruit, but they are strongly deactivated during the critical pressing step, probably because of oxidation phenomena [103]. So, the replacement of these enzymes is expected to be appropriate in relation with the role they play in determining the final product quality [100].

### 2.9. Carotenoid Extraction

Carotenoids are the main group of coloring substances in nature being responsible for many plant colors from red to yellow [104]. There is a continuously growing market for carotenoids as food colorants due to their desirable properties, such as their natural origin, null toxicity, and high versatility, providing both lipo- and hydrosoluble colorants with colors ranging from yellow to red [104]. In addition, provitamin A activity, a role in lipid oxidation, and anticarcinogenic properties are very important biological functions of these pigments [104].

Usually a combination of cellulolytic and pectinolytic enzymes accelerates the rate of hydrolysis for achieving complete liquefaction. Cellulase randomly splits cellulose chains into glucose whereas commercial pectinase preparations from *Aspergillus niger *have pectinesterase (PE), polygalacturonase (PG), and pectin lyase (PL) activity [104, 105]. The use of pectinase and cellulase enzymes disrupts the cell wall of orange peel, sweet potato and carrot, and releases the carotenoids in the chloroplasts and in cell fluids. These pigments remain in their natural state still bound with proteins. This bonded structure prevents pigment oxidation and also affects color stability [104, 106], whereas solvent extraction dissociates the pigments from the proteins and causes water insolubility and ease of oxidation [104, 107].

### 2.10. Detergent Industry

Use of cellulases along with protease and lipase in the detergents is a more recent innovation in this industry [17]. Cellulase preparations capable of modifying cellulose fibrils can improve color brightness, feel, and dirt removal from the cotton blend garments. The industrial application of alkaline cellulases as a potential detergent additive is being actively pursued with a view to selectively contact the cellulose within the interior of fibers and remove soil in the interfibril spaces in the presence of the more conventional detergent ingredients [5, 17]. Nowadays, liquid laundry detergent containing anionic or nonionic surfactant, citric acid or a water-soluble salt, protease, cellulose, and a mixture of propanediol and boric acid or its derivative has been used to improve the stability of cellulases. As most of the cellulose fibers in the modern textile industry enzymes are used increasingly in the finishing of fabrics and clothes are arranged as long, straight chains of some small fibers can protrude from the yarn or fabric. The cellulases are applied to remove these rough protuberances for a smoother, glossier, and brighter-colored fabric [37].

### 2.11. Waste Management

The wastes generated from forests, agricultural fields, and agroindustries contain a large amount of unutilized or underutilized cellulose, causing environmental pollution [108, 109]. Nowadays, these so-called wastes are judiciously utilized to produce valuable products such as enzymes, sugars, biofuels, chemicals, cheap energy sources for fermentation, improved animal feeds, and human nutrients [6, 13, 37, 44, 69, 110, 111].

## 3. Conclusions

The biological aspects of processing of cellulosic biomass become the crux of future research involving cellulases and cellulolytic microorganisms. Cellulases are being commercially produced by several industries globally and are widely being used in food, animal feed, fermentation, agriculture, pulp and paper, and textile applications. With modern biotechnology tools, especially in the area of microbial genetics, novel enzymes and new enzyme applications will become available for the various industries. Improvements in cellulase activities or imparting of desired features to enzymes by protein engineering are probably other areas where cellulase research has to advance.

## Figures and Tables

**Table 1:** Microorganisms having cellulolytic abilities.

Fungi	Soft rot fungi
	*Aspergillus niger; A. nidulans; A. oryzae; A. terreus; Fusarium solani; F. oxysporum; Humicola insolens; H. grisea; Melanocarpus albomyces; Penicillium brasilianum; P. occitanis; P. decumbans; Trichoderma reesei; T. longibrachiatum; T. harzianum; Chaetomium cellulyticum; C. thermophilum; Neurospora crassa; P. fumigosum; Thermoascus aurantiacus; Mucor circinelloides; P. janthinellum; Paecilomyces inflatus; P. echinulatum; Trichoderma atroviride*
	Brown rot fungi
	*Coniophora puteana; Lanzites trabeum; Poria placenta; Tyromyces palustris; Fomitopsis sp.*
	White rot fungi
	*Phanerochaete chrysosporium; Sporotrichum thermophile; Trametes versicolor; Agaricus arvensis; Pleurotus ostreatus; Phlebia gigantea*
Bacteria	Aerobic bacteria
	*Acinetobacter junii; A. amitratus; Acidothermus cellulolyticus; Anoxybacillus sp.; Bacillus subtilis; B. pumilus; B. amyloliquefaciens; B. licheniformis; B. circulan; B. flexus; Bacteriodes sp.; Cellulomonas biazotea; Cellvibrio gilvus; Eubacterium cellulosolvens; Geobacillus sp.; Microbispora bispora; Paenibacillus curdlanolyticus*; *Pseudomonas cellulosa; Salinivibrio sp.; Rhodothermus marinus *
	Anaerobic bacteria
	*Acetivibrio cellulolyticus; Butyrivibrio fibrisolvens; Clostridium thermocellum; C. cellulolyticum; C. acetobutylium; C. papyrosolvens; Fibrobacter succinogenes; Ruminococcus albus*

Actinomycetes	*Cellulomonas fimi; C. bioazotea; C. uda; Streptomyces drozdowiczii; S. lividans; Thermomonospora fusca; T. curvata*

**Table 2:** Applications of cellulases in various industries.

Industry	Applications
Agriculture	Plant pathogen and disease control; generation of plant and fungal protoplasts; enhanced seed germination and improved root system; enhanced plant growth and flowering; improved soil quality; reduced dependence on mineral fertilizers

Bioconversion	Conversion of cellulosic materials to ethanol, other solvents, organic acids and single cell protein, and lipids; production of energy-rich animal feed; improved nutritional quality of animal feed; improved ruminant performance; improved feed digestion and absorption; preservation of high quality fodder

Detergents	Cellulase-based detergents; superior cleaning action without damaging fibers; improved color brightness and dirt removal; remove of rough protuberances in cotton fabrics; antiredeposition of ink particles

Fermentation	Improved malting and mashing; improved pressing and color extraction of grapes; improved aroma of wines; improved primary fermentation and quality of beer; improved viscosity and filterability of wort; improved must clarification in wine production; improved filtration rate and wine stability

Food	Release of the antioxidants from fruit and vegetable pomace; improvement of yields in starch and protein extraction; improved maceration, pressing, and color extraction of fruits and vegetables; clarification of fruit juices; improved texture and quality of bakery products; improved viscosity fruit purees; improved texture, flavor, aroma, and volatile properties of fruits and vegetables; controlled bitterness of citrus fruits

Pulp and Paper	Coadditive in pulp bleaching; biomechanical pulping; improved draining; enzymatic deinking; reduced energy requirement; reduced chlorine requirement; improved fiber brightness, strength properties, and pulp freeness and cleanliness; improved drainage in paper mills; production of biodegradable cardboard, paper towels, and sanitary paper

Textile	Biostoning of jeans; biopolishing of textile fibers; improved fabrics quality; improved absorbance property of fibers; softening of garments; improved stability of cellulosic fabrics; removal of excess dye from fabrics; restoration of colour brightness

Others	Improved carotenoids extraction; improved oxidation and colour stability of carotenoids; improved olive oil extraction; improved malaxation of olive paste; improved quality of olive oil; reduced risk of biomass waste; production of hybrid molecules; production of designer cellulosomes
